# Editorial: Code Case - Investigating Transparency and Reproducibility

**DOI:** 10.1523/ENEURO.0233-17.2017

**Published:** 2017-08-10

**Authors:** Christophe Bernard

Dear friends and colleagues,

Everyone from scientists, reviewers, to funding agencies and publishers, talks about the lack of reproducibility and transparency in present research. Many high-profile papers are based on highly specialized techniques that few have mastered, which make them very difficult to reproduce. Many laboratories have developed their own software to perform data analysis. These are rarely evaluated by reviewers, although they should be since they also constitute a proper result of the study and contribute significantly to the findings. It is conceivable that many studies cannot be reproduced because they are underpowered (the mythical *n* = 5). But it is surprising to find that many computational studies and modeling papers cannot be reproduced either. After all, they are based on mathematics, and mathematics cannot lie, can they?

There are many reasons why computational studies and modeling papers cannot be reproduced, including variations of initial conditions from one computer to another, different motherboard clocks, etc. Nevertheless, these results should be reproducible, and there should be a way to test routines and algorithms designed to process data.

In order to ascertain the validity of the results and conclusions, reviewers should have access to codes to run simulations, test algorithms, etc. But sometimes the authors do not provide the codes, even when papers are published.

At *eNeuro*, we are taking steps to solve the issue of transparency and reproducibility in computational work and modeling papers, by asking authors to include the code they have used to obtain the results in their published paper. We will recommend that authors deposit their code in a suitable repository such as GitHub, ModelDB, BioModels, CellML, or Visiome and then include the accession numbers in their manuscript. The authors are free to update the code in repositories, but not the code linked to the paper on *eNeuro*’s website. We will also require the data to be available upon acceptance, and availability is maintained after the publication of the manuscript. Thus, other scientists will be able to try to reproduce the results of the paper.

In addition, we strongly recommend authors include any analysis software they have designed. This recommendation may become a requirement in the future.

With this new initiative, our goal is to improve transparency and reproducibility. The new policy is appended below.

As always, feel free to send me comments, suggestions, etc. to build a better science environment at eneuroeditor@sfn.org.

Cheers,

Policy on Computer Code and Software

***Computational models***: New computational neuroscience computer code must be submitted included with the submission as Extended Data and be deposited in a suitable repository such as GitHub, ModelDB, BioModels, CellML, or Visiome. Studies using custom code deemed central to the conclusions must include a statement in the Materials and Methods section, under the heading “Code Accessibility,” indicating whether and how the code can be accessed, including any accession numbers or restrictions and be cited in the references. Code must be available upon acceptance and publication of the manuscript. No code is to be withdrawn following publication. Code files must be packaged in a single ZIP file.

***Software***: If new software or a new algorithm is used for data analysis, authors are encouraged to include the software or algorithm with the submission as Extended Data and deposit it in an appropriate public repository. A statement needs to be included in the Materials and Methods section, under the heading “Software Accessibility,” indicating whether and how the software or algorithm can be accessed, including any accession numbers or restrictions. Software files must be packaged in a single ZIP file.

